# The Influence of Heel Height on Strain Variation of Plantar Fascia During High Heel Shoes Walking-Combined Musculoskeletal Modeling and Finite Element Analysis

**DOI:** 10.3389/fbioe.2021.791238

**Published:** 2021-12-20

**Authors:** Meizi Wang, Shudong Li, Ee-Chon Teo, Gusztáv Fekete, Yaodong Gu

**Affiliations:** ^1^ Faculty of Sports Science, Ningbo University, Ningbo, China; ^2^ Faculty of Health and Safety, Óbuda University, Budapest, Hungary; ^3^ School of Mechanical and Aerospace Engineering, Nanyang Technological University, Singapore, Singapore; ^4^ Savaria Institute of Technology, Eötvös Loránd University, Budapest, Hungary

**Keywords:** high heel shoes, finite element model, musculoskeletal modeling, plantar fascia, plantar fasciitis

## Abstract

The therapeutic benefit of high heel shoes (HHS) for plantar fasciitis treatment is controversial. It has been suggested that plantar fascia strain can be decreased by heel elevation of shoes which helps in body weight redistribution throughout the length of the foot. Yet it is a fact that the repetitive tension caused by HHS wearing resulting in plantar fasciitis is a high-risk disease in HHS individuals who suffer heel and plantar pain. To explore the biomechanical function on plantar fascia under HHS conditions, in this study, musculoskeletal modeling (MsM) and finite element method (FEM) were used to investigate the effect of heel height on strain distribution of plantar fascia. Three-dimensional (3D) and one-dimensional (1D) finite element models of plantar fascia were generated to analyze the computed strain variation in 3-, 5-, and 7-cm heel heights. For validation, the computed foot contact pressure was compared with experimental measurement, and the strain value on 1D fascia was compared with previous studies. Results showed that the peak strain of plantar fascia was progressively increased on both 3D and 1D plantar fascia as heel elevated from 3 to 7 cm, and the maximum strain of plantar fascia occurs near the heel pain site at second peak stance. The 3D fascia model predicted a higher strain magnitude than that of 1D and provided a more reliable strain distribution on the plantar fascia. It is concluded that HHS with narrow heel support could pose a high risk on plantar fasciitis development, rather than reducing symptoms. Therefore, the heel elevation as a treatment recommendation for plantar fasciitis is questionable. Further studies of different heel support structures of shoes to quantify the effectiveness of heel elevation on the load-bearing mechanism of plantar fascia are recommended.

## 1 Introduction

The plantar fascia is a rather complex and important structure, which has different biomechanical functions in gait, such as supporting transverse and longitudinal arch, facilitating force transmission between foot and ground, balancing weight-bearing distribution on foot, cushioning ground reaction force, and preventing the foot from injury (D’Ambrogi et al., 2003; Wearing et al., 2007; Mahowald et al., 2011; Stecco et al., 2013; Chen et al., 2015). However, any injury of plantar fascia inevitably affects the biomechanical function of the foot. In the United States, approximately two million people experience symptoms of heel and plantar pain due to plantar fascia injury yearly (Chen et al., 2015). Known as plantar fasciitis, it is caused by excessive repetitive loading of the plantar fascia leading to microtears and inflammation of the calcaneal adhesions (Luffy et al., 2018; Trojian and Tucker, 2019), and it is a common complaint among women, especially those wearing high heel shoes (HHS) regularly (López-López et al., 2018).

It is evidence that wearing HHS adversely affects the musculoskeletal system, altering ankle–foot complex function, changing the force transmission pattern of muscle tendon, and interfering in load distribution of the foot (Di Sipio et al., 2018; Wiedemeijer and Otten, 2018; Wan et al., 2019). The length of the calcaneus to metatarsals is shortened, accompanied by arch rising under HHS conditions, leading to a change in arch morphology, transferring a greater portion of weight-bearing to the forefoot, and reducing the plantar contact area of the midfoot, leading to increased contraction and tension force on the plantar fascia (Sun et al., 2017). Khodair and Younes investigated the relationship between the plantar fascial pathology and HHS wearing in 40 female patients with resulting heel pain; they reported that 30 patients had fascial edema at calcaneal insertion and the plantar fascia thickened; the signal intensity in plantar fascia increased in 21 patients, which are characteristic signals of plantar fasciitis (Khodair and Younes, 2019).

On the contrary, there are different opinions about the effect of HHS on plantar fascia strain. Some investigators have suggested that the appropriate heel elevation was beneficial in the treatment of plantar fasciitis on account of the plantar fascia tension could be temporarily reduced by heel raising (Kogler et al., 2001; Marshall, 1988). It was believed that the angle discrepancy between the hindfoot and metatarsus decreased under HHS, which allowed more movement of the forefoot in the plantar-flexion position and reduced the fascia tension (Kogler et al., 2001). Recently, Yu et al. investigated the influence of heel height on strain stress on the plantar fascia using finite element method (FEM); its total tension force was reduced by 77.3% as heel height rose from 0 to 5.08 cm during balanced standing (Yu et al., 2016). However, the plantar fascia in this research was simply represented by the 1D linear truss element, which may overestimate its elastic modulus resulting in an inaccurate plantar fascia strain (Erdemir et al., 2004; Chen et al., 2015).

Accordingly, to address the controversial views of tension force in plantar fascia as the main crucial biomechanical concern in foot pathology in the HHS population to further understand the influence of the heel height on plantar fascia tension, a solid 3D plantar fascia modeling is established. In this study, the effects of HHS of three different heel heights (3, 5, 7 cm) on the strain–stress variation of 3D plantar fascia were observed by using a combination of FEM and musculoskeletal modeling. A comparison of 3D and 1D digitization of the plantar fascia, with 1D of plantar fascia created by linear truss elements that simply connect the heel and five proximal phalanxes, was carried out.

## 2 Methods

In this study, a healthy female (age: 26 years old; height: 165 cm; weight: 53 kg) with no sign of musculoskeletal pathology and lower-limb injury was recruited. For data acquisition, gait capturing 3D motions of the subject wearing HHS in three different heel heights (3, 5, 7 cm) and each gait corresponding relevant muscle forces and strain distribution in plantar fascia were computed using musculoskeletal modeling (MsM) and FEM, respectively.

**FIGURE 1:**
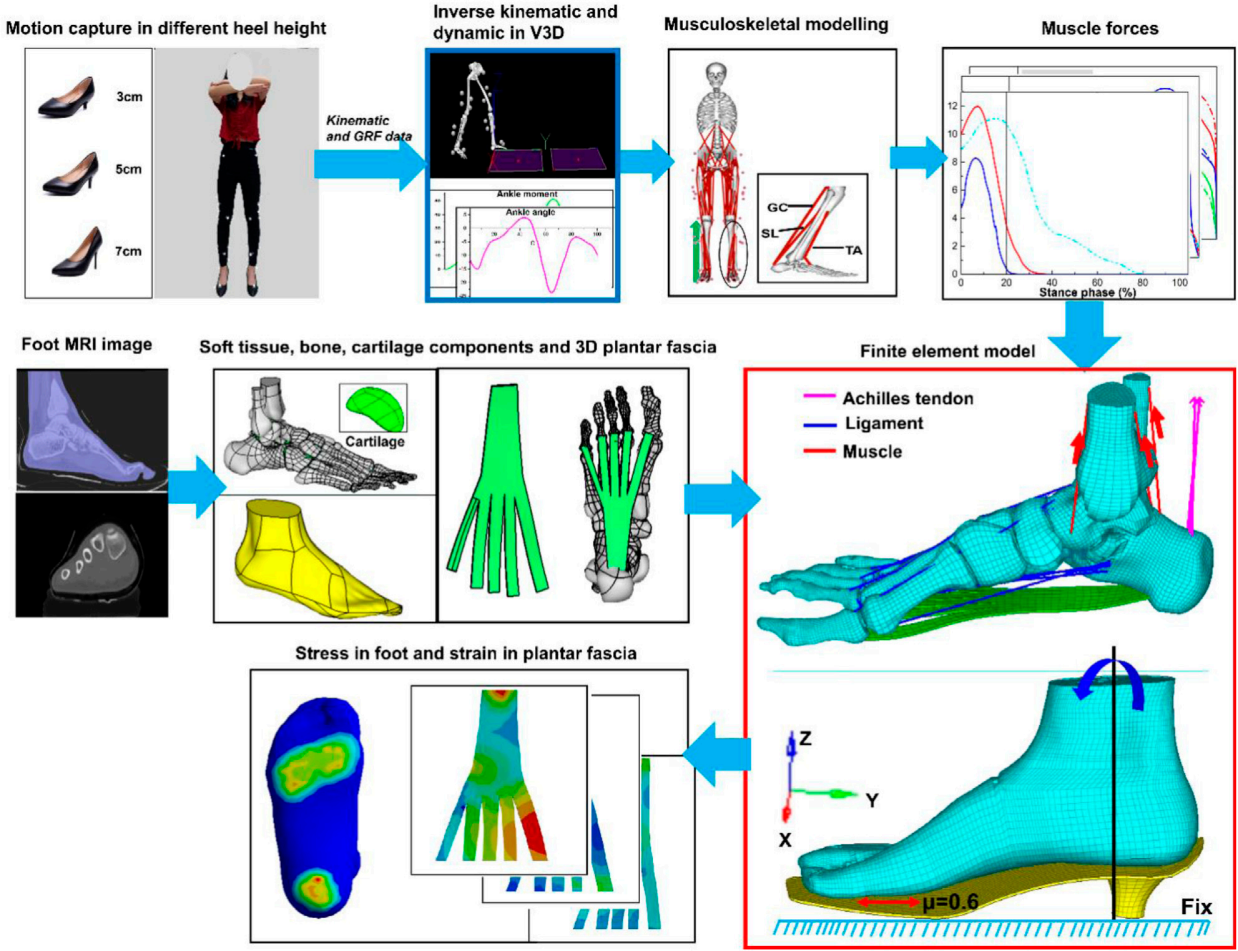
The workflow of the study.

The protocol was approved by Ningbo University following the declaration of the ethical committee. The subject has informed all contents regarding the test with written consent before the experiment. The workflow of the study is shown in Figure 1.

### 2.1 Gait Analysis

The gait analysis was used to drive MsM and FEM. A Vicon motion capture system (Oxford Metrics Ltd., Oxford, UK) consisting of eight cameras and two AMTI force platforms (Watertown, MA, USA) was utilized to capture the kinematic and kinetic lower extremities during HHS gait. The kinematic and kinetic data were measured synchronously at frequencies of 100 and 1,000 Hz, respectively. The marker set was placed on the subject’s various lower-extremity key points according to generic GaitModel 2,392 in Opensim. One static standing trial and six successful waking trials were captured on each heel height condition. For walking trail data acquisition, the subject walked through the motion capture area in a straight direction at their self-pace with both feet stepping entirely at the force platforms. The captured representative data of six trials of each heel height were selected for MsM analysis.

### 2.2 MsM Analysis

To calculate the relevant muscle forces in the lower limbs, GaitModel 2,392 as a generic MsM was selected in Opensim. GaitModel 2,392 has 10 main rigid body segments, 23 degrees of freedom, and 92 musculotendon actuators to represent 76 muscles in the lower limbs and torso (Kainz et al., 2016). Three major extrinsic muscle groups of lower extremities—gastrocnemius (GC), soleus (SL), and tibialis anterior (TA)—for foot movement were digitized. GC and SL play important roles in ankle plantar flexor, and TA produces major dorsiflexion moment to the ankle during gait (Hwang et al., 2006).

**FIGURE 2:**
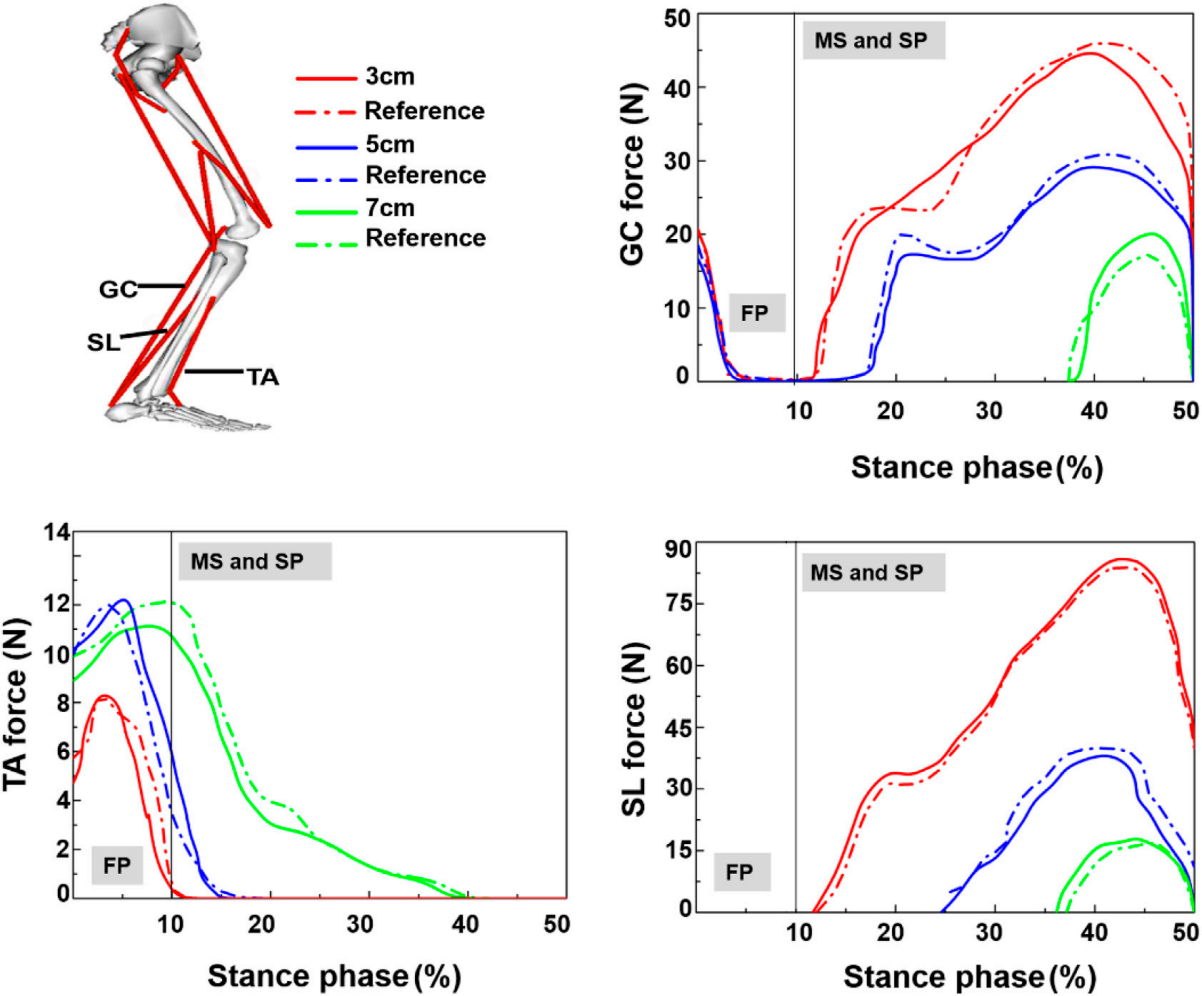
Muscle force in stance phase, TA = tibialis anterior; GC = gastrocnemius; SL = soleus; FP = first peak; MS = mid-standing; SP = second peak (Hwang et al., 2006; Son et al., 2008).

The kinematic data and ground reaction force (GRF) in 3-, 5-, and 7-cm heel height conditions were converted into Opensim from the V3D to calculate the muscle forces. A three-step process was performed in Opensim: firstly, the selected MsM was scaled to accommodate the height and mass of the test subject with adjusting muscle attachments and length. Then, the kinematic data was loaded into the Opensim gait model. Secondly, the residual reduction algorithm was applied to match the coordinates of the model more dynamically consistent with the measured GRF and movement which drive the generalized coordination of the dynamic musculoskeletal model toward the desired kinematic trajectory. Finally, the computed muscle control algorithm was applied to calculate muscle activations and muscle forces in three heel height conditions. These peak muscle forces obtained from Opensim were validated against electromyography (EMG) data acquired from the previous studies (Figure 2) (Hwang et al., 2006; Son et al., 2008), and these validated muscle forces were assigned as boundary and loading conditions for the FEM analysis.

### 2.3 FEM Analysis

#### 2.3.1 Model Construction and Material Property

MRI scan was performed on one subject using a 3.0-T Siemens MRI system; the foot ankle was fixed at a neutral position using a brace during the CT scan, and the images of the sagittal and coronal plane were collected (repetition time: 4.11 s; slice thickness: 1 mm; and matrix: 320 × 260). The geometrical shapes of the right foot model were segmented based on magnetic resonance images of the participant using Mimics 18.0 (Materialise, Leuven, Belgium). The 28 anatomical segmented bones and one encapsulated soft tissue together with cartilages were generated based on areas of two adjacent segmented bones using SolidWorks (SolidWorks Corporation, MA, USA) to create the solid model. The muscles and 1D plantar fascia were created in Hypermesh. Three muscle groups GC, TA, and SL and 20 ligaments and Achilles tendon together with the plantar fascia connecting the corresponding points between the calcaneal tubercle and five proximal phalanges were modeled as 1D linear truss elements. The 1D plantar fascia and ligament elements were given a cross-sectional area to present strain response. For the 3D plantar fascia, a thickness of 2 mm was generated in SolidWorks according to the anatomical atlas (Hedrick, 1996). The FEM of HHS based on the subject’s shoe size (37 EU size) of 3-, 5-, and 7-cm heel height shoes were generated in SolidWorks, respectively. Table 1 shows the material property of shoes, bone, muscle, plantar fascia, cartilage, ligament, Achilles tendon, and encapsulated soft tissue, as previously reported (Lemmon et al., 1997; Pailler-Mattei et al., 2008; Siegler et al., 1988; Gu et al., 2010a). All elements were idealized as linearly elastic materials, except that the encapsulated soft tissue was defined as hyperelastic material properties (Gu et al., 2010b).

**TABLE 1:** Material properties of the components in the finite element model.

	Elastic modules (MPa)	Poisson ratio	Cross section (mm^2^)	Mass density ρ (kg/m^3^)
Bulk soft tissue	Second-order polynomial strain hyperelastic model (C_10_ = 0.8556, C_01_ = 0.05841, C_20_ = 0.03900, C_11_ = 0.02319, C_02_ = 0.00851, D_1_ = 3.65273)		—	
Bone	7,300	0.30	—	1,500
Sole	200,000	0.42	—	7,800
Ligaments	260	—	18.4	-
Cartilage	1	0.40	—	1,050
3D plantar fascia	350	0.45	—	—
1D plantar fascia	350	—	58.6	—

#### 2.3.2 Boundary and Loading Condition

The loading condition during walking in FEM simulation was determined from experimental data of the participant. Three instants [the GRF first peak (25% stance phase), the GRF valley (45% stance phase), and the GRF second peak (60% stance phase)] of the stance phase were used to drive the foot model.

**FIGURE 3:**
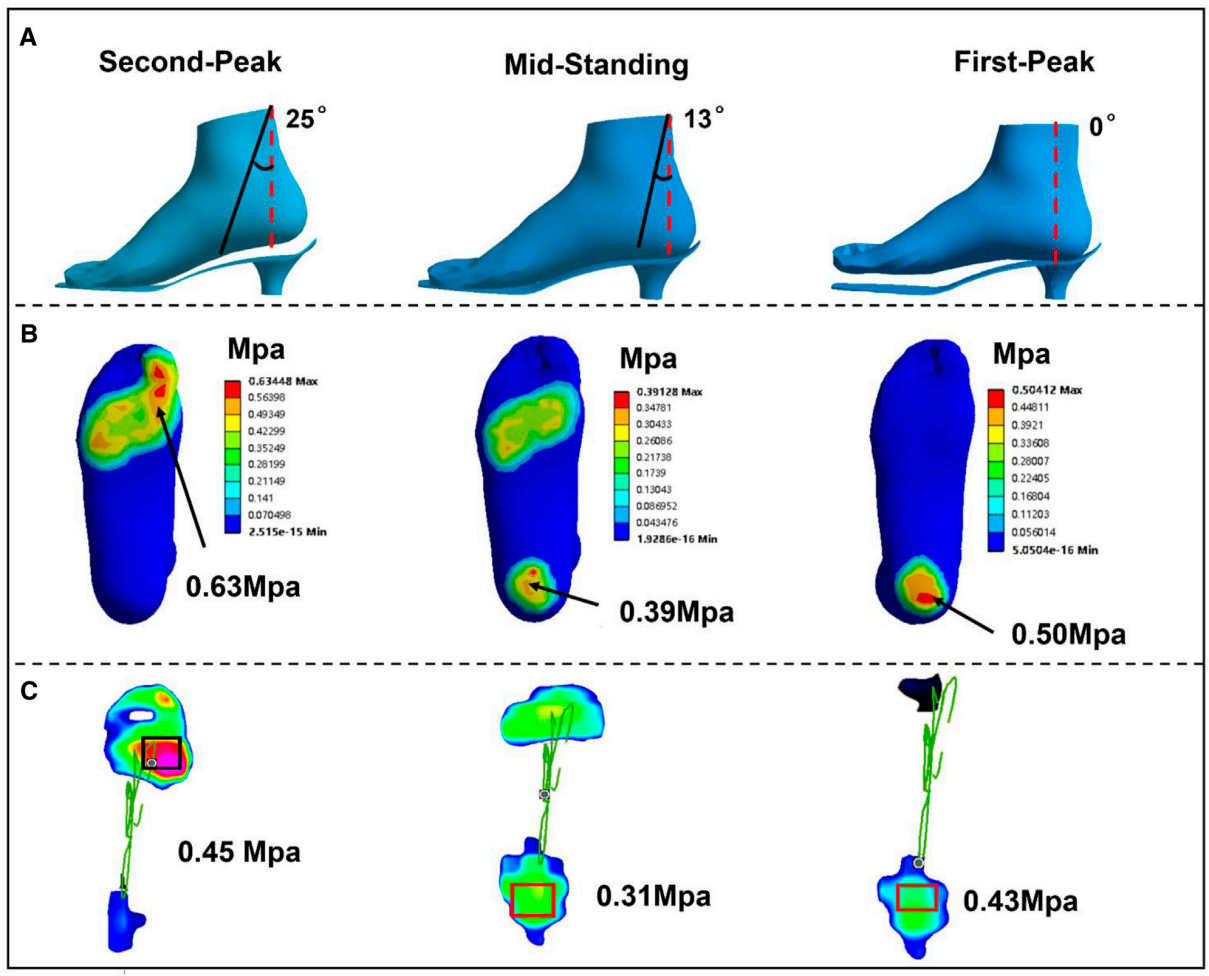
**(A)** The different stance phase of the foot relative to the sole of shoes during simulation in 3-cm heel height. **(B)** FEM predicted. **(C)** Plantar contact pressure in experimental measurement.

The generated foot solid model comprised of the foot bony components and 3D plastic fascia embedded in the encapsulated soft tissue. Prior to the simulation runs, with the sole of shoes fully restrained, the foot model was placed as heel strike position with an angle between the plantar and sole set at 20°, which was the angle acquired in experimental data between the sole of the shoe and the ground. With the tibia set as the axis of rotation defined by the load joint option in Ansys (ANSYS, Inc., Canonsburg, PA, USA), an inclination angle of the tibia relative to the ground of 0° was set as the initial first peak stance condition. The tibia was further rotated about the *X*-axis from 0° to 25° relative to the vertical ground axis (Figure 3A) to define the mid-standing stance and second-peak stance conditions, and the rotation moment function described by ankle moment in the sagittal plane. In the analysis, the foot FEM model translation and rotation in coronal and transverse planes were not considered. For the surface between the sole and the forefoot, a friction coefficient of 0.6 was defined (Yu et al., 2013), and the respective GC, SL, and TA force acquired from the MsM (Figure 2) at three stances were applied, with Achilles tendon forces obtained from previous research applied (Yu et al., 2013). The Transient Solver was selected in Ansys to avoid convergence problems.

#### 2.3.3 Geometry Mesh

The foot model was predominately meshed with hexahedral elements with less than 5% of tetrahedral elements in Hypermesh (14.0). The irregular bony and soft tissue parts were sectioned and re-meshed manually to improve the quality of the mesh with an aspect ratio of 3D element close to 1 and Jacobi ratio of 0.6 (Li et al., 2017). Simple loading and boundary conditions were applied to investigate the mesh sensitivity of the whole foot structure based on computed results of the soft tissue, bony component, location of plantar in the soft tissue, anterior and posterior ends of the plantar fascia, and the connection region between bones and cartilages in Ansys. The element sizes were gradually reduced until the variation of force displacement is less than 3% between the two size meshes (Li et al., 2017), with the final model consisting of 279,037 elements.

#### 2.3.4 FEM Validation

The validation was conducted by comparing the plantar pressure of the experimental measurements and FEM predictions in the three different stance phases of three different heel height conditions. Firstly, the plantar pressure was measured by an in-shoe system (Novel Pedar-X System, Novel GmbH, Munich, Germany, www.novel.de) during the HHS gait in each heel height condition with each corresponding peak contact pressure recorded. The plantar insole in this study was selected based on the shoe size as well as foot size of the subject to minimize the extent of deformation, while the pressure insole was calibrated before the test to reduce the error. Secondly, both experimental and predicted maximum contact pressures in nine plantar regions (big toe, other toes, three equally divided regions in the forefoot, lateral and medial of midfoot and rearfoot) were collected. Then by applying the Pearson correlation, |r|, the agreement between predictions and experimental measurements of 18 data pairs was compared. Values of |r| ≤ 0.35, 0.36 ≤ |r| ≤ 0.67, and 0.68 ≤ |r| ≤ 1.0 represent weak, moderate, and strong correlation, respectively (Taylor, 1990). The predicted tension of the 1D plantar fascia was compared with existing research, as well as muscle force in GC, SL, and TA.

## 3 Results

### 3.1 Validation

Figures 3B,C show the plantar pressure distribution of the 3-cm heel height condition obtained from the FEM prediction and the experimental measurement in three stance phases. Correlation analysis showed a high linear relationship between the FEM prediction and measurement (r = 0.83; confidence interval: 95%, 0.57–0.90; *p* < 0.002). The differences between the experimentally measured and computationally estimated values are 14% in the first peak, 20.5% in mid-standing, and 28.6% in the second peak. Figure 2 shows the muscle forces calculated by Opensim compared with previous studies; the graphs show similar corridor trends (Son et al., 2008; Hwang et al., 2006). Lastly, the variation range of predicted strain in 3D and 1D plantar fascia models at mid-standing is 3%–5.7%, which was consistent with previous measurements (Kogler et al., 2001). Based on these validation results, our simulation results are considered to be reliable.

### 3.2 Changing of the Strain Value on Plantar Fascia Among Different Heel Heights

**FIGURE 4:**
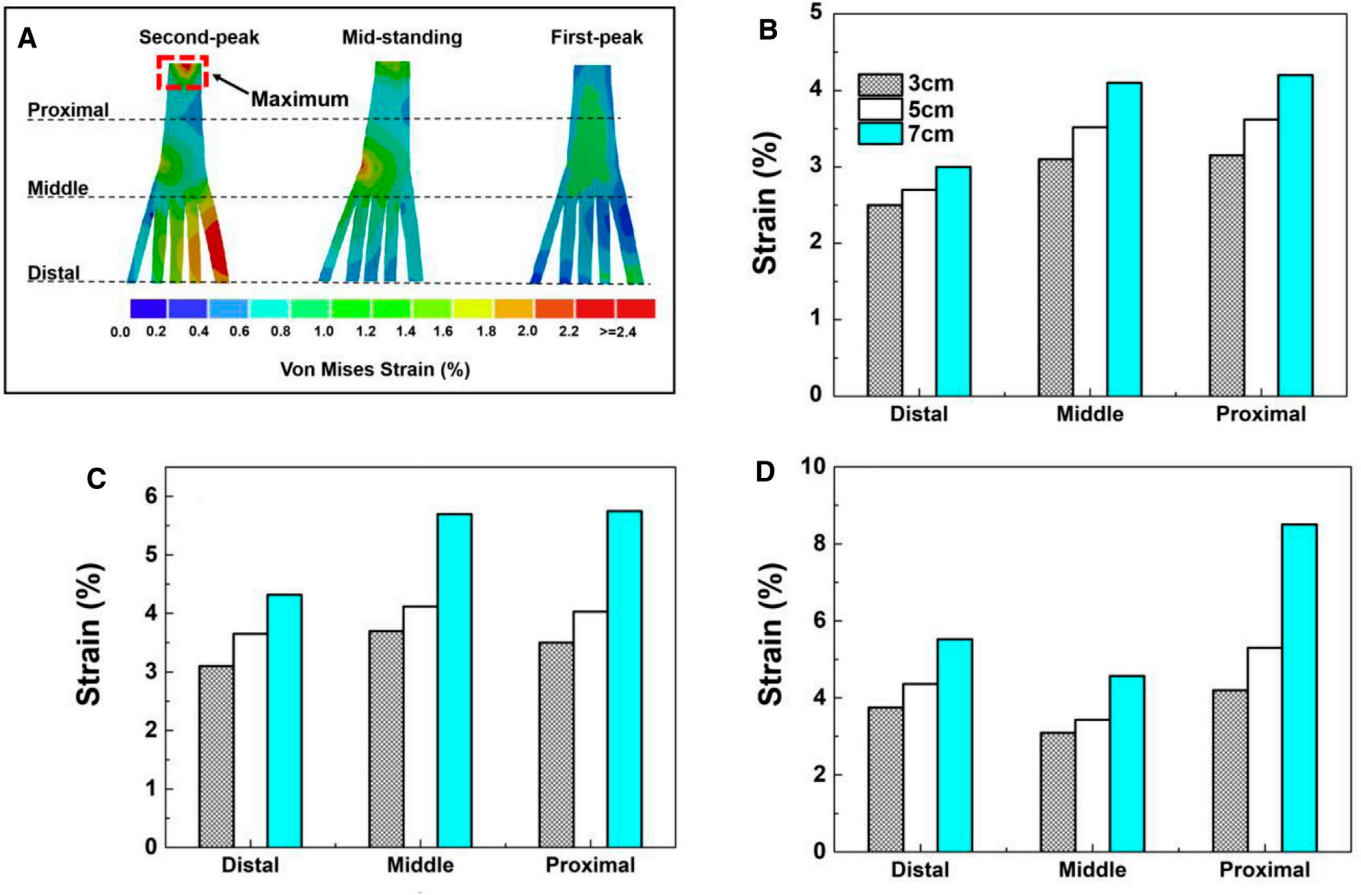
**(A)** The peak 3D fascia strain for three heel heights in three gait events. **(B)** First-peak phase. **(C)** Mid-standing phase. **(D)** Second-peak phase.

As predicted, strain distributions of plantar fascia from FEM simulations showed similar patterns for three different heel heights in three different stances; Figure 4A shows the typical strain distribution pattern on the plantar fascia in the 5-cm heel height condition. The two end portions of the 3D plantar fascia tied to the bones at the proximal and distal regions were excluded to avoid excessive deformation, and the remaining region of the 3D fascia was divided equally into proximal, medial, and distal regions. FEM prediction results showed that heel height had a significant effect on the strain level of fascia in three stance phases.

For the strain level of plantar fascia, irrespective of heel heights, at the first peak and mid-standing phases, the middle and proximal regions experience a similar level of higher peak strain on the fascia than in the distal region. At the second-peak phase, the plantar fascia strain presents the highest value in the proximal region, followed by the distal region with the lowest strain in the middle region.

For different heel heights, the simulations predicted different plantar fascia strain distributions at different regions irrespective of stance phases. Figure 4D shows that the highest plantar fascia strain occurred in the second peak stance phase. In this stance phase, in the proximal region, the peak strain increased by 102% for heel heights of 3–7 cm. For heel heights raised from 3 to 5 cm, the fascia strain increased by 26.1%, and there is a sharp increase by 60.3% from 5 to 7 cm of heel height.

However, for the model with fascia modeled as 1D truss elements, there are no distinct three regions as defined for 3D fascia. The peak strain in 1D fascia is smaller than that in 3D fascia in all three different heel heights in all different stance phases.

## 4 Discussion

Due to the complex interaction between foot movement and internal components, it is difficult to investigate the biomechanical mechanism of the plantar fascia *in vitro* measurements. Therefore, establishing a realistic plantar fascia simulation method is the premise to understand the biomechanical response of plantar fascia under HHS conditions. In this study, a combination method of FEM and MsM was used to evaluate the strain characteristics of the plantar fascia during HHS walking in three different heel heights.

The opinions on heel elevation as a treatment approach for plantar fasciitis are questionable based on the results of this study. The plantar fascia strain progressively increased in both 3D and 1D modeling when heel height was elevated from 3 to 7 cm. The highest strain was found at the 7-cm heel height, the lowest was found at the 3-cm heel height, and there was a sharp increase by 60.3% from 5 to 7 cm of heel height. However, the findings differ from previous reports (Yu et al., 2016). Yu et al. indicated that the average tension force and strain on the plantar fascia decreased from 151.0 N (strain: 0.74%) to 59.6 N (strain: 0.28%), which reduced by 60.5% and 62.2%, respectively, when the heel height elevated from 0 to 5.08 cm during balanced standing with HHS, then there was a remarkably increase from 5.08 to 7.62 cm of heel height. They suggested that the strain of the plantar fascia could be decreased by an appropriate heel height. According to Yu et al., the 1D element of the plantar fascia was used in their simulation; this simplification did not take into account the complex relationship between ligaments and articular surfaces. In terms of foot structural complexity, the biomechanical response of the plantar fascia under loading is different in the lateral and medial aspects, and it may be responsible for limiting the predicted loading result in an abnormal plantar fascia strain when it is depicted as a 1D truss (Hedrick, 1996). Therefore, a more comprehensive 3D modeling could provide insight into the biomechanical response of the plantar fascia variation under HHS conditions. In our study, 3D fascia modeling shows a higher average strain than that of 1D fascia at all heel heights, and the strain variation of the entire 3D fascia structure can be demonstrated. The peak strain of the plantar fascia in the proximal region was larger than that of the middle and distal portions in three different heel heights, especially at the second peak phase. The results were consistent with previous reports that stress and strain were concentrated on the medial calcaneus tubercle, close to the site of heel pain (Peng et al., 2021). However, this strain response does not appear in the current 1D fascia model or the previous linear fascia model.

Meanwhile, the predicted results of this study are different from previous cadaver experiments, in which plantar fascia strain decreased gradually from a heel height of 2–6 cm (Kogler et al., 2001). According to cadaver investigation, the heel elevation was achieved by a contoured platform with a shank profile located in the mid-region, simulates the inner weight-bearing surface of shoes, and provides complete support for the foot arch. The decreased plantar fascia strain was induced by an extended shank profile from under the calcaneus to the cuboid; this may be enough to relieve severe symptoms of plantar fasciitis (Kogler et al., 2001). Researchers have suggested that the load transmission pattern could be changed by shoe interface configuration, further affecting the strain response of the plantar fascia (Alexander et al., 1990; Kogler et al., 2001; Speksnijder et al., 2005). Differently, in our study, the stiletto shoes with a narrow base support were used which could lead to plantar pressure concentrating on the hindfoot and forefoot as well as reduce the contact area on the mid-foot (Chevalier et al., 2010). Furthermore, the arch is raised passively and reached its limit of flattening with a toe flexion that requires greater muscle force to keep body posture stability under stiletto shoe conditions. However, maintaining the posture under HHS conditions demands a higher-level activity on intrinsic muscles that becomes inadequate, especially in terms of a long period using HHS that muscles are easily prone to fatigue (Mika et al., 2011). Thus, the plantar fascia is more likely to experience tightness and obtain a higher tension force. The appropriate heel elevation may contribute to a strain relief on the plantar fascia, but it certainly does not refer to HHS with a narrow heel base support. In further research, the influence of different sole structures of shoes on plantar fascia strain and tension force should be investigated.

On the other hand, the peak strain on the plantar fascia reaches the maximum at the second peak stance compared with the first peak and valley stance phase. Under HHS conditions, the metatarsophalangeal joints (MPJ) are kept in a dorsiflexion angle which progressively increased with heel elevation. Healey and Chen demonstrated that the increased MPJ dorsiflexion as a result of the elevated hindfoot is a compensation mechanism to stabilize the body posture (Healey and Chen, 2010). However, the larger dorsiflexion angle of the toes could increase the tension force on the plantar fascia due to the “windlass mechanism,” which has been well described by Hicks in 1954 (Hicks, 1954). During the push-off phase, the toes are forced into an extended position as toe-standing; the plantar pad is pulled forward, then it moves the attached fascia anterior, contributing a relatively shorter longitudinal arch. Therefore, the plantar fascia will be subjected to a greater tension force when the forefoot makes a push on the ground during the gait. In a cadaver model test, Erdemir et al. observed that plantar fascia tension gradually increased during stance and reached the maximum at the late stance phase (Erdemir et al., 2004).

Certain limitations in this research should be discussed. Firstly, the realistic simulation of the mechanical behavior of the plantar fascia is important for plantar fascia biomechanics response and effect of pathological conditions (Behforootan et al., 2017). However, the material properties of ligament, cartilages, and plantar fascia were idealized as linearly elastic, in order to compare simulation results with the previous one (Yu et al., 2016); thus, the same material properties were adopted and the hyperelastic or viscoelastic behavior was ignored here. Although this consideration could reduce computational costs and complexity, the simplified material properties may negatively affect internal strain distribution on plantar fascia. As for how different material models may affect the strain distribution of the plantar fascia in specific applications, such as HHS conditions, further research is needed. Secondly, only extrinsic muscle forces were involved in this simulation, while other intrinsic muscles were not considered. Similar to the flexor digitorum brevis muscle that was longitudinally adhered by the central plantar fascia, its absence may adversely affect the accuracy of the fascia strain value. Thirdly, FEM analysis is always limited to a specific subject and does not take into account differences in individual foot morphology. Lastly, the more the ModelGait 2,392 we used here limits DOFs at the ankle joint, the more detailed should be the foot model used in future research to evaluate the foot function related to pathology.

## 5 Conclusion

A 3D FEM model of plantar fascia has been established to explore the effect of heel elevation on plantar fascia internal loading during HHS walking, and a 1D plantar fascia model was created for comparison. The results from the present research could reveal the strain variation on the plantar fascia and facilitate understanding of potential causes of plantar fasciitis induced by using HHS. According to this study, 3D plantar fascia could reflect the entire biomechanical change on the plantar fascia than 1D and provide a reliable strain distribution on the plantar fascia under HHS conditions. The strain on the plantar fascia in this study was progressively increased instead of reduced as heel height rose from 3 to 7 cm. The trend of increased strain on the plantar fascia under HHS conditions is more in line with the “windlass mechanism.” Meanwhile, the higher strain on the proximal region of the fascia provides evidence for plantar fasciitis development in the HHS population. Proper heel elevation may help to relieve plantar fascia tension, but it certainly does not refer to HHS with narrow heel support. Therefore, the HHS as a treatment recommendation for plantar fasciitis is questionable. Considering the foot morphology as a determinant factor in load transmission patterns that can be shaped by footwear, the variation sole structure of shoes should be investigated in the future to quantify the effect of different force transmission patterns on the plantar fascia.

## Data Availability

The original contributions presented in the study are included in the article/supplementary material; further inquiries can be directed to the corresponding authors.
